# The chemokine receptor CXCR4 regulates satellite cell activation, early expansion, and self-renewal, in response to skeletal muscle injury

**DOI:** 10.3389/fcell.2022.949532

**Published:** 2022-09-22

**Authors:** Ahmed S. Shams, Robert W. Arpke, Micah D. Gearhart, Johannes Weiblen, Ben Mai, David Oyler, Darko Bosnakovski, Omayma M. Mahmoud, Gamal M. Hassan, Michael Kyba

**Affiliations:** ^1^ Lillehei Heart Institute, Minneapolis, MN, United States; ^2^ Department of Pediatrics, University of Minnesota, Minneapolis, MN, United States; ^3^ Department of Human Anatomy and Embryology, Faculty of Medicine, Suez Canal University, Ismailia, Egypt; ^4^ Department of Genetics, Cell Biology and Development, University of Minnesota, Minneapolis, MN, United States

**Keywords:** satellite cells, skeletal muscle, activation, regeneration, CXCR4

## Abstract

Acute skeletal muscle injury is followed by satellite cell activation, proliferation, and differentiation to replace damaged fibers with newly regenerated muscle fibers, processes that involve satellite cell interactions with various niche signals. Here we show that satellite cell specific deletion of the chemokine receptor CXCR4, followed by suppression of recombination escapers, leads to defects in regeneration and satellite cell pool repopulation in both the transplantation and *in situ* injury contexts. Mechanistically, we show that endothelial cells and FAPs express the gene for the ligand, SDF1α, and that CXCR4 is principally required for proper activation and for transit through the first cell division, and to a lesser extent the later cell divisions. In the absence of CXCR4, gene expression in quiescent satellite cells is not severely disrupted, but in activated satellite cells a subset of genes normally induced by activation fail to upregulate normally. These data demonstrate that CXCR4 signaling is essential to normal early activation, proliferation, and self-renewal of satellite cells.

## Introduction

Skeletal muscle is a highly dynamic tissue with a remarkable capacity for rapid regeneration following injury. The essential precursors driving this regenerative process are the satellite cells, classified as a group of mononuclear, self-renewing, and tissue-resident stem cells accounting about 2%–5% of the mononuclear cells of skeletal muscle (Asakura et al., 2002; Bosnakovski et al., 2008; Shadrach and Wagers, 2011; Brack and Rando, 2012). During homeostasis, satellite cells are classically defined by their unique location between the sarcolemma and basal lamina of multinucleated myofibers and by their expression of the paired homeobox protein PAX7 (Mauro 1961; Seale et al., 2000; Bosnakovski et al., 2008; Yin et al., 2013). The satellite cell pool is heterogenous, and has been described to express numerous surface markers including vascular cell adhesion molecule (VCAM-1), Integrin α7 (ITGA7), neural cell adhesion molecule (NCAM1), hepatocyte growth factor receptor, also known as c-MET, and m-Cadherin (Cornelison et al., 2001; Fukada et al., 2004; Ieronimakis et al., 2010; Biressi et al., 2013; Liu et al., 2015). Various markers have been used either independently or in combination to isolate the satellite cell population, and among those frequently used is the chemokine receptor CXCR4 (Sherwood et al., 2004; Sacco et al., 2008; Conboy et al., 2010; Chapman et al., 2013; Yoshida et al., 2013; Stitelman et al., 2014; Gromova et al., 2015; Barruet et al., 2020). CXCR4 is present on most cells of the satellite cell pool, including more than 94% of Pax7-ZsGreen positive cells, which express a fluorescent reporter marking PAX7 expression that can be used to isolate fresh satellite cells from muscle digest (Maesner et al., 2016; Arpke et al., 2021). CXCR4 has also been documented on some satellite cell derived cell lines (Ratajczak et al., 2003; Vasyutina et al., 2005). Outside of skeletal muscle, CXCR4 is expressed in numerous types of blood cells, and in the central nervous system (Jazin et al., 1997; Moepps et al., 1997).

The CXCR4 receptor, when activated by its ligand CXCL12, also known as stromal cell-derived factor 1 (SDF-1), plays a crucial role in cell migration during inflammation and organogenesis (Cencioni et al., 2012; Zhao et al., 2014). It can mediate migration of resting leukocytes and hematopoietic progenitors (Janowski 2009; Monzel et al., 2018). Furthermore, CXCR4/SDF1 signaling is important for the migration of primordial germ cells, a function that is conserved in fish, birds, and mammals (Doitsidou et al., 2002; Knaut et al., 2003; Molyneaux et al., 2003; Stebler et al., 2004). CXCR4 or SDF1 mutations have been shown to affect the migration of cerebellar granule cells, hippocampal, and cortical neuronal progenitors (Lazarini et al., 2003). In addition to the regulation of various migration processes, CXCR4/SDF1 also controls growth and survival of various cell types (Molyneaux et al., 2003; Bianchi and Mezzapelle 2020).

During embryogenesis, the SDF1/CXCR4 axis has a major role in skeletal muscle development, particularly with regard to migration of myogenic progenitors. Population of the limb buds (Odemis et al., 2005; Yusuf et al., 2006), tongue, and facial muscles (Yahya et al., 2020) with myogenic progenitors have all been demonstrated to be dependent on CXCR4. SDF-1 is expressed in the mesenchyme of the limb and the first branchial arch, which represent targets of the migrating cells and the application of SDF-1 implants into the limb of chick embryos redirects the muscle progenitor cells toward the ectopic source of the factor and inhibits their differentiation (Vasyutina et al., 2005). In addition, *Cxcr4* mutants mice show a significant increase in the number of apoptotic muscle progenitors, suggesting that *Cxcr4* signals provide not only attractive cues but also control survival (Chong et al., 2007; Bryson-Richardson and Currie 2008).

In adult skeletal muscle tissue the role of CXCR4 signaling in skeletal muscle physiology is less well defined. It has been shown that fibroblasts secrete SDF-1 influencing hematopoietic cells in muscle (Ratajczak et al., 2003), leading to the activation of signaling pathways which stimulate satellite cell migration to sites of inflammation or required regeneration (Ratajczak et al., 2003; Relaix et al., 2021). A recent study has suggested a role for CXCR4 in protecting the satellite cells against the inflammatory induced damage during muscle regeneration (Lahmann et al., 2021). While these studies suggest potential mechanisms by which CXCR4 signaling has an impact on muscle regeneration, well-defined mechanisms whereby CXCR4 regulates satellite cell function are yet to be determined. We therefore designed this study to address the *in vivo* necessity of CXCR4 in satellite cells. By investigating satellite cell maintenance, self-renewal, engraftment after transplantation, and muscle regeneration in the context of satellite cell-specific temporally-regulated genetic ablation of the CXCR4 receptor, we demonstrate a specific role for *Cxcr4* in the activation and early stages of proliferation of satellite cells.

## Materials and methods

### Mice

All animal experiments in this study were performed in accordance with protocols approved by the Institutional Animal Care and Use Committee at the University of Minnesota. We combined the *Cxcr4* ^*fl*^ (008,767 B6.129P2-Cxcr4^tm2Yzo^/J Jackson Laboratories), and *Pax7*-ZsGreen (Bosnakovski et al., 2008) alleles with either the *Pax7*-creERT2 (Keefe et al., 2015) (kindly provided by Gabrielle Kardon) or the *Ubc*-creERT2 (007,001 B6. Cg-^Ndor1Tg(UBC−cre/ERT2)1Ejb^/1J Jackson Laboratories) alleles to generate both *Cxcr4* ^*FL/FL*^ *; Pax7-creERT2; Pax7-ZsGreen* mice and *Cxcr4 FL/FL; Ubc-creERT2; Pax7-ZsGreen* mice. All experiments were conducted using adult mice 3–4 months of age. Transplant recipients were *NSG-mdx* ^*4Cv*^ mice (Arpke et al., 2013).

For *Cxcr4* conditional knockouts, all mice were treated for five consecutive days with 80 mg/kg IP tamoxifen (Sigma Aldrich) dissolved at a concentration of 85 mg per 1 ml 100% ethanol then mixed with sunflower oil (Sigma Aldrich) to reach a final concentration of 12.75 mg in 1 ml vehicle, after which mice were then maintained on tamoxifen diet (TD.140251 Envigo) for the remaining duration of each experiment. For the transplantation experiments, donor mice were not treated with tamoxifen, however recipient mice were treated starting on the day of transplantation.

### Satellite cell harvest

Isolation of bulk satellite cells from hind limbs was performed as described previously (Arpke et al., 2013; Arpke and Kyba 2016). Briefly, hind limb muscles were carefully dissected and cut longitudinally with a razor blade parallel to the muscle fibers, using forceps each time pressing the blade to separate the fibers. The minced muscle was incubated shaking for 75 min in 0.2% collagenase type II (Gibco, Grand Island, NY) in high glucose Dulbecco’s modified Eagle’s medium (DMEM) containing 4 mM L-glutamine 4,500 mg/L glucose, and sodium pyruvate (HyClone, Logan, UT) supplemented with 1% Pen/Strep (Gibco) at 37°C. Samples were washed twice with Rinsing Solution (F-10+), Ham’s/F-10 medium (HyClone) supplemented with 10% Horse Serum (HyClone), 1% 1 M HEPES buffer solution (Gibco), and 1% Pen/Strep. Samples were centrifuged at 500 G for 5 min. After aspiration of supernatant, the sample was resuspended in F-10 containing collagenase type II and dispase (Gibco), vortexed, and incubated shaking at 37°C for 30 min. Samples were vortexed again, drawn and released into a 10 ml syringe with a 16-gauge needle four times, then with a 18-gauge needle four times to release the cells from the muscle fibers prior to passing the cell suspension through a 40-µm cell strainer (Falcon, Hanover Park, IL, United States). The sample was drawn and released into a 10 ml syringe with the 18-gauge needle four additional times and passed through a new 40-µm cell strainer. Samples were centrifugated for 5 min 500 G at 4°C, and resuspended in Fluorescent-activated Cell Sorting (FACS) staining medium: Phosphate Buffered Saline (PBS, Corning, Manassas, VA, United States) containing 2% fetal bovine serum (HyClone) and 0.5 μg/ml propidium iodide, for FACS analysis and sorting on a FACSAriaII (BD Biosciences, San Diego, CA, United States).

Quantification of satellite cells from single injured or control TA muscles was performed similarly. Digested muscle samples were drawn and expelled into a 3 ml syringe four times with 18-gauge needle. The cell suspension was passed through a 40-μm cell strainer. 3 ml of F-10+ was added to each sample to prevent over-digestion; and samples were centrifuged, then resuspended in FACS staining medium. For transplanted TAs, the samples were stained using an antibody mixture of PE-Cy7 rat anti-mouse CD31, PE-Cy7 rat anti-mouse CD45, Biotin rat anti-mouse CD106 (VCAM) and PE Streptavidin from BD Biosciences; and ITGA7 647. Suppliers and clonal identifiers are provided in Supplementary Table S1. The number of donor (ZsGreen^+^) satellite cells and total satellite cells (lineage negative; VCAM, ITGA7 double positive cells) was determined by running the entire volume through the FACS and recording all events. The TA samples were resuspended in 200 µl FACS staining medium (PBS with 2% FBS and propidium iodide) then the total volume was run out on a BD FACS Aria II, with red (641 nm), blue (488 nm) and yellow-green (561 nm) lasers. Propidium iodide-negative (live) cells were gated into either PE (empty) vs. ZsGreen for unstained samples, or for stained samples: APC (ITGA7) vs. PE-Cy7 (Lin), gating Lin-neg cells into APC (ITGA7) vs. PE (VCAM), gating double-positive cells into SSC vs. ZsGreen and counting ZsGreen+ cells. In order to detect the number of CXCR4+ cells within the ZsGreen+ population, the muscle digest was stained with Biotin rat anti-mouse CD184 (CXCR4) monoclonal antibody followed by Streptavidin-PE. In order to isolate the different niche cells we digested uninjured and 48 h post-CTX injury quadriceps muscles as previously described and stained the mononuclear cells with the following antibodies: FITC rat anti-mouse CD31, PE-Cy7 rat anti-mouse CD45, ITGA7 647 and PE rat anti-mouse CD140A (PDGFRα) (Bosnakovski et al., 2020).

### Colony forming cell assay

Satellite cells were identified by FACS and single cell-sorted into 96-well plates in mouse myogenic medium (MMM): DMEM/F12 medium without L-glutamine (Cell Gro, Manassas, VA; 15-090-CV) containing 20% FBS (HyClone), 10% horse serum (Gibco, 26050-088), 50 ng/μl human basic fibroblast growth factor (Peprotech, Rocky Hill, NJ; 100-18), 1% penicillin/streptomycin (Gibco, 15140-122), 1% Glutamax (Gibco, 35050-061), and 0.5% chick embryonic extract (US Biological, Swampscott, MA; C3999). Plates were maintained at 37°C at 5% CO_2_, 5% O_2_, 90% N_2_) for 8 days. Colonies were identified and fixed with 4% paraformaldehyde for 20 min at room temperature, stained with MF20 myosin heavy chain antibody (Developmental Studies Hybridoma Bank, University of Iowa) and counterstained with DAPI and imaged on a Zeiss AxioObserver Z1 inverted microscope with an AxioCamMR3 camera (Thornwood, NY, United States) (Ippolito et al., 2012).

### TA injury and transplantation

Adult (3–4 months old) *Cxcr4* ^FL/FL^; *Pax7*-ZsGreen mice carrying *Pax7*-creERT2 or not were anesthetized with ketamine and xylazine, both hind limbs shaved and sterilized using surgical betadine solution, the skin over the TA was opened with a scissor and both TA muscles were exposed. 25 µl cardiotoxin (10 µM in PBS, Sigma, Saint Louis, MO, United States) was injected with a Hamilton syringe and the skin was closed using nonabsorbable suture. For transplantation experiments, 48 h prior to transplantation of cells, 4 month-old NSG-mdx^4Cv^ mice were anesthetized with ketamine and xylazine and both hind limbs were subjected to 1,200 cGy irradiation using an RS 2000 Biological Research Irradiator (Rad Source Technologies, Inc., Suwanee, GA) with lead shields protecting the body and forelimbs. 24 h prior to transplant, cardiotoxin injury was performed as above. 24 h after this, 300 ZsGreen satellite cells were collected by FACS from donor mice and transplanted in a volume of 10 µl PBS into each TA. 4 weeks after transplantation, one transplanted TA of each mouse was harvested and prepared for sectioning and staining as described previously (Arpke et al., 2013), while the other transplanted TA was prepared for FACS analysis as described above.

### Histology and immunohistochemistry for dystrophin and/or laminin

TA muscles were removed and placed in OCT Compound (Scigen Scientific, Gardena, CA, United States), frozen in liquid nitrogen-cooled 2-methylbutane (Sigma) and stored at −80°C. 10 µm cryosections were cut on a Leica CM3050 S cryostat (Leica Microsystems, Buffalo Grove, IL, United States). Cryosections were air dried then stained with H&E, Sirius Red/Fast Green (SR/FG) or processed for immunohistochemistry. Briefly, slides were fixed with 4% paraformaldehyde, washed 3 times with PBS, permeabilized with 0.3% Triton X-100 (sigma) and blocked for 1 h with 3% BSA in permeabilization solution. Slides were incubated overnight at 4°C with a rabbit polyclonal antibody to dystrophin and/or a mouse monoclonal antibody to laminin (antibody details in Supplementary Table S1), then sections were incubated for 90 min at RT with Alexa Fluor 555 goat anti-rabbit and/or Alexa Fluor 488 goat anti-mouse IgG antibodies (Life Technologies, Grand Island, NY, United States). Coverslips were mounted with Immu-Mount (Thermo Scientific, Kalamazoo, MI, United States). Slides were imaged with a Zeiss Axio Imager. M2 with an AxioCam MRm camera (Carl Zeiss Microscopy, LLC, Thornwood, NY, United States). Images from slides stained with laminin only were used to measure the fiber CSA, while in case of laminin and dystrophin co-staining the number of donor muscle fibers was determined by manually counting dystrophin+ fibers.

The percent fibrosis in the TA was quantified on SR/FG-stained TA sections using ImageJ software. The image was first converted to grayscale based on green, then the threshold was adjusted. The area-stained red was measured and normalized to cross-sectional area (CSA) (Bosnakovski et al., 2022).

### RNA extraction, cDNA synthesis, RTqPCR and transcriptional profiling

Satellite cells were sorted directly into the RNA lysis buffer and the RNA was extracted using the protocol provided with the RNeasy Mini Kit (Qiagen). cDNA was synthesized using High-Capacity cDNA Reverse Transcription Kit (Applied Biosystems). Efficiency of *Cxcr4 in vivo* deletion was determined with Mm01292123_m1 *Cxcr4* TaqMan probe (ThermoFisher). To harvest the RNA from cultured myoblasts, medium was removed and RNA lysis buffer was applied directly over the adherent cell layer then. Lysate was processed as described by the manufacturer.

For RNA-seq, total RNA from freshly sorted and overnight cultured *Pax7*-ZsGreen^+^ cells from both hind limbs of *Cxcr4* WT and *Cxcr4* KO mice was isolated as above. Total RNA samples were submitted to Genewiz (NJ, United States) for quality control analysis, library generation and sequencing using the Illumina HiSeq 2 × 150 paired-end configuration. The raw sequencing data (FASTQ files) with approximately 20 million reads per sample were adapter trimmed (TrimGalore 0.6.0) and mapped to the GRCm38. p6/mm10 genome with STAR (2.7.2a). The number of reads mapping to GENCODE M25 gene annotations were quantified with Rsubread (2.2.6). Differential gene expression was determined using DESeq2 (1.34.0). Genes were ranked by the Wald test-statistic for gene set enrichment analysis (GSEA 4.1.0) using the top 500 up- and down-regulated genes upon satellite cell activation as previously defined in (Machado et al., 2017). Figures were prepared with ggplot2 (3.3.5). Sequence data is deposited in GEO (GSE205015).

### Measuring time to first division

*Pax7*-ZsGreen cells were isolated from *Cxcr4* ^FL/FL^, *Ubc*-creERT2 wild type or mutant mice by FACS and plated for live-cell imaging into 0.1% gelatin-coated 24-well glass-bottom dishes (NC9988706; Mattek; Thermo Fisher Scientific; Waltham, MA, United States) (7,000 cells/well) containing myoblast growth medium, MGM: Ham’s F10 medium with L-glutamine (HyClone) containing 20% FBS (HyClone) and 1% Glutamax (Gibco, 35050-061) 1% Pen/Strep (Gibco) and 10 ng/ml hb-FGF (PeproTech, Cranbury, NJ, United States). Cells from the two sources were treated with MGM containing 5 mM 4-hydroxy tamoxifen (4OHT) at the time of plating and provided fresh medium 18 h after plating. Time-lapse imaging was performed from 18 to 52 h after plating with a Nikon Eclipse Ti-inverted fluorescence microscope equipped with an automated stage (Prior) and a custom chamber to maintain a constant 37°C temperature, high humidity, and 5% CO_2_. Multiple positions were analyzed per group with images acquired every 10 min using phase contrast. Images were collected using a 20X CFI Plan Apochromat Lambda (NA = 0.75) objective (Nikon). For each condition, at least 100 individual cells were tracked. Following imaging, data were exported as individual TIFFs for each position and time point. ImageJ was used to concatenate TIFF images from each location and time to first division was determined for each as previously described (Larson et al., 2022).

### *In vitro* EdU proliferation assay

*Pax7*-ZsGreen cells were isolated by FACS and plated into 0.1% gelatin-coated 96-well plates (1,000 cells/well) containing MGM. 4OHT (HelloBio) dissolved in DMSO was applied daily (5 mM final concentration). At day 7, we added EdU 10 µM (Invitrogen) for 6 h, fixed cells with 4% PFA. Cells were stained with Click-iT EdU Cell Proliferation Kit for Imaging, Alexa FluorTM 555 dye (C10338; Invitrogen) according to manufacturer’s instructions. The cells were then incubated in 4,6-diamidino-2-phenylindole (DAPI 1:1,000 dilution in PBS) for 20 min at room temperature. EdU+ nuclei were identified and imaged at 10X magnification on a Zeiss Observer. Z1 inverted microscope equipped with an AxioCam Mrm camera (Thornwood, NY, United States).

### ATP cell proliferation luciferase assay

*Pax7*-ZsGreen cells were isolated by FACS and plated into 0.1% gelatin-coated 96-well plates (1,000 cells/well) containing MGM with or without 5 mM 4OHT. At day 7 post-plating, medium was replaced with CellTitre-Glo (G7570; Promega, Madison, WI) reagent (1:3) in 100 µl of PBS. Plates were allowed to equilibrate for 3 min, then read on a Cytation3 plate reader (BioTek, Winooski, VT, United States).

### Statistical analysis

All data were analyzed with two-tailed unpaired Student *t*-tests for determining significant differences between two groups or one-way ANOVA with Tukey’s post hoc for determining significant differences between three or more groups; data was tested for normality using (Kolmogorov–Smirnov test). Significance was considered at *p* values of <0.05. Data are presented as mean ± SEM unless otherwise indicated. All statistical testing was performed using GraphPad Prism 8.0 (GraphPad Software Inc., San Diego, CA, United States). Sample sizes are reported as the number of independent mice from which the cells were analyzed or isolated. All histology images were processed and analyzed in a blinded manner with samples being de-identified as to study or control.

## Results

### CXCR4 is necessary for repopulation of the satellite cell pool upon transplantation

**FIGURE 1:**
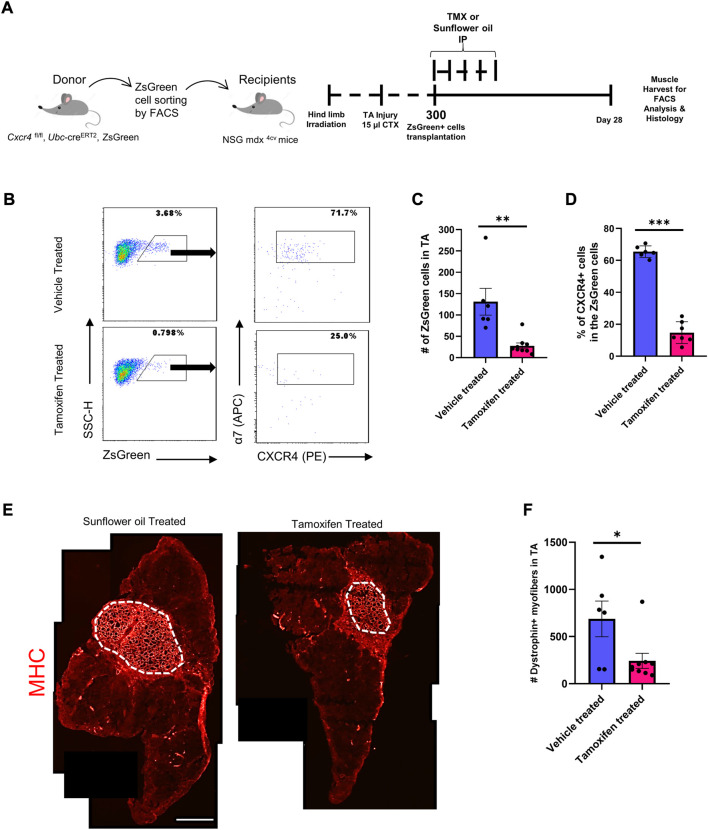
*Cxcr4* Knockout impairs engraftment and repopulation of the satellite cell pool upon transplantation. **(A)** Schematic for the transplantation assay. **(B)** Representative FACS plots from both the TMX- and vehicle-treated groups showing the number of the ZsGreen^+^ cells in the TA muscle after transplantation and the percent of CXCR4^+^ cells among the ZsGreen^+^ ITGA7^+^ cell fraction. **(C)** Total number of ZsGreen^+^ cells in TA muscles of recipient NSG-mdx4cv mice. Vehicle treated (n = 6). Tamoxifen treated (*n* = 9). **(D)** Percentage of the CXCR4^+^ population in the ZsGreen^+^ cells in recipient TA muscles. **(E)** Representative immunofluorescence images for TA muscles stained by Dystrophin (Red). Scale bar = 100 µm. **(F)** Total number of Dystrophin^+^ fibers in the TA muscles of vehicle and tamoxifen treated recipient mice. Vehicle treated (*n* = 6). Tamoxifen-treated (*n* = 9). Data are presented as mean ± SEM,*p < 0.05, **p < 0.01 and ***p < 0.001 by *t*-test.

We previously developed a transplantation-based assay to monitor self-renewal and differentiation potential of satellite cells in which 300 Pax7-ZsGreen (Bosnakovski et al., 2008) cells are transplanted bilaterally into injured, irradiated tibialis anterior muscles of NSG-mdx^4Cv^ mice (Arpke et al., 2021). One month after transplant, one TA muscle is digested and run completely through a flow cytometer to quantify contribution to the satellite cell pool while the other TA muscle is used for dystrophin immunostaining to quantify contribution to the differentiated fiber population. We adapted this assay to test the role of CXCR4 in these processes by using donor *Pax7*-ZsGreen mice that also carried homozygous floxed *Cxcr4* alleles and the strong ubiquitously expressed *Ubc-creERT2*. Although *Ubc-creERT2* is expressed in every cell lineage enabling conditional knockout of *Cxcr4* in all cell types (Supplementary Figure S1B), because only satellite cells are transplanted, treatment of the recipient mice with tamoxifen deletes *Cxcr4* only in the donor cells. Half of the recipients received tamoxifen to delete *Cxcr4* while half received vehicle, starting on the day of transplant (Figure 1A). FACS analysis demonstrated that the *Cxcr4* knockout resulted in a tremendous inhibition of satellite cell self-renewal evident by a 3-fold drop in the number of *Pax7*-ZsGreen cells in the recipient TA muscles of the TMX-treated group (Figures 1B,C, Supplementary Figure S1A). To assess the *Cxcr4* deletion efficiency, we stained the TA muscle digest for cell surface receptors ITGA7 and CXCR4 and determined the frequency of CXCR4^+^ cells among the ITGA7^+^ ZsGreen^+^ cells, which revealed a dramatic reduction of CXCR4^+^ cells in the TMX-treated group, in those rare cells that populated the satellite cell compartment 1 month post-transplant (Figures 1B,D).

We then evaluated the differentiation efficiency of the *Cxcr4*-deleted satellite cells by counting dystrophin+ (donor-derived) fibers. This revealed a significant drop in the number of dystrophin+ fibers in the TA sections of the TMX-treated group compared to the vehicle treated (Figures 1E,F), indicating that absence of *Cxcr4* impaired both the repopulation of the satellite pool as well as the regeneration of new fibers.

We repeated these transplants using a design in which all mice were TMX-treated, but were transplanted with donor cells from mice either carrying or lacking creERT2. For these studies, we used the *Pax7-creERT2* allele (Keefe et al., 2015) which is satellite cell-specific but expresses at somewhat lower efficiency than *Ubc*-creERT2. To suppress revertants, after the course of TMX injections, recipients were kept on TMX chow. These conditions replicated results with *Ubc*-creERT2 (Supplementary Figures S1C–E).

To evaluate the efficiency of *in vivo* deletion under steady state conditions prior to injury, we isolated hind limb satellite cells immediately after the course of five consecutive doses of TMX IP, and evaluated *Cxcr4* expression on *Pax7*-ZsGreen cells by FACS and RTqPCR spanning the deleted exon 2. While *Cxcr4* mRNA was reduced by 90%, protein expression levels on satellite cells were reduced more modestly (Supplementary Figures S2B–D), likely reflecting the fact that protein turnover is low in quiescent cells and suggesting that steady state changes might take longer timeframes to identify.

### CXCR4 enables expansion of the satellite cell pool after injury

**FIGURE 2:**
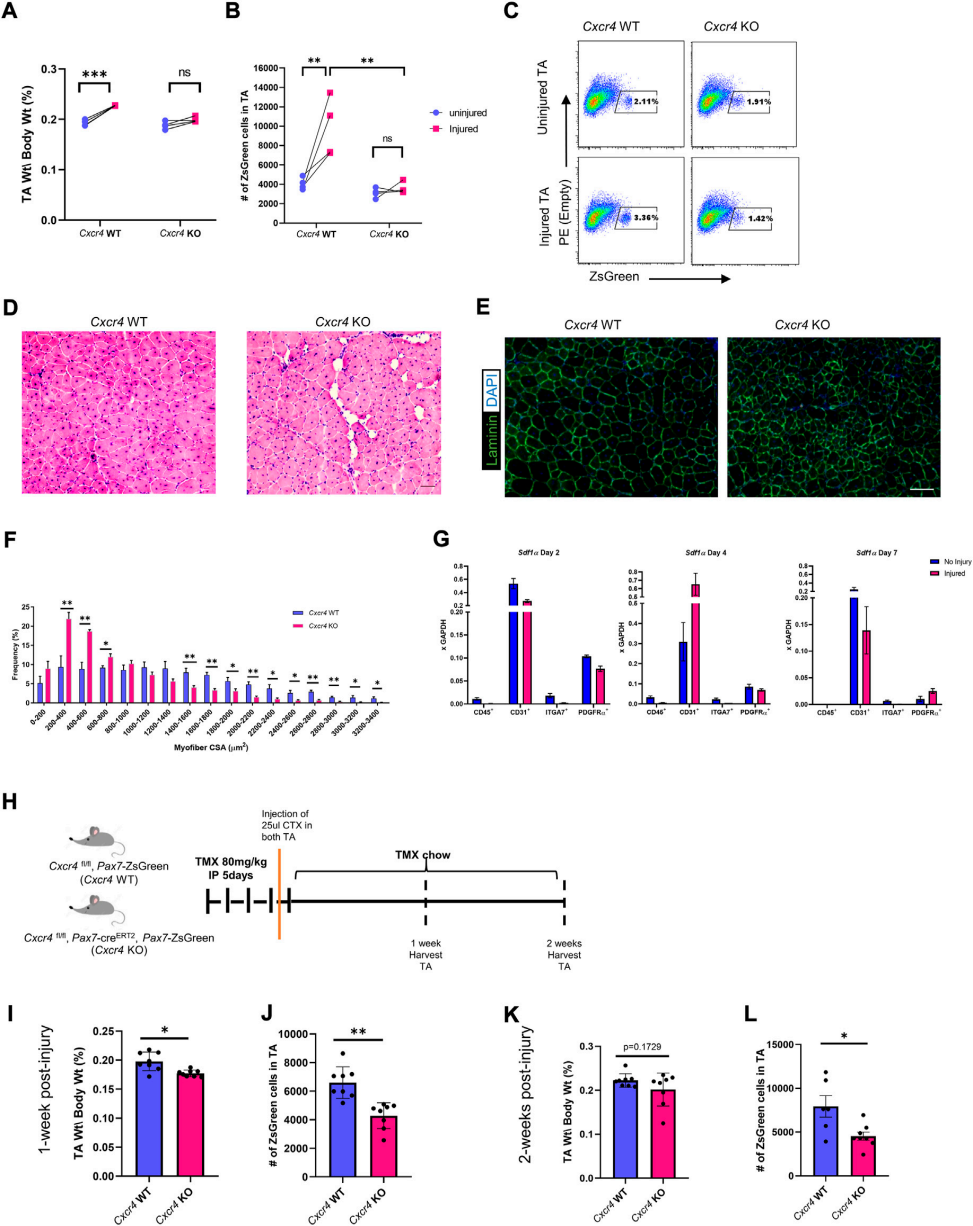
CXCR4 is necessary for proper skeletal muscle regeneration and expansion of the satellite cell pool after injury. **(A)** TA mass normalized to body weight in uninjured and injured, *Cxcr4* WT and KO, mice. **(B)** Total number of ZsGreen^+^ cells in the TA muscle of uninjured and injured, *Cxcr4* WT (*n* = 4) and KO (*n* = 4), mice. **(C)** Representative FACS plots showing the total number of ZsGreen^+^ cells in uninjured and injured, *Cxcr4* WT (*n* = 4) and KO (*n* = 4), mice. **(D)** Representative H&E staining of TA sections from *Cxcr4* WT and KO mice 4 weeks after the second injury. Scale bar = 50 µm. **(E)** Representative IF staining for laminin (green) and DAPI (blue) in TA sections from *Cxcr4* WT and KO mice 4 weeks after the second injury. Scale bar = 100 µm. **(F)** Myofiber cross sectional area distribution in TA muscles of *Cxcr4* WT (*n* = 5) and KO (*n* = 5), 4 weeks after the second injury. **(G)** RTqPCR for *Sdf1a* in hematopoietic (CD45^+^), endothelial (CD31^+^), myogenic (ITGA7^+^), and FAP cells (PDGFRα^+^) in uninjured and 2, 4, 7 days post-injury muscles (*n* = 4) mice. **(H)** Schematic for the short-term (1 and 2 weeks) post-injury studies. **(I)** TA mass normalized to body weight in *Cxcr4* WT and KO mice 1 week post CTX injury. **(J)** Total number of ZsGreen^+^ cells in the TA muscle of *Cxcr4* WT (*n* = 4) and KO (*n* = 4), mice 1 week post CTX injury. **(K)** TA mass normalized to body weight in *Cxcr4* WT and KO mice 2 weeks post CTX injury. **(L)** Total number of ZsGreen^+^ cells in the TA muscle of *Cxcr4* WT (*n* = 4) and KO (*n* = 4), mice 2 weeks post CTX injury. Data are presented as mean ± SEM,*p < 0.05, **p < 0.01 and ***p < 0.001 by *t*-test and two-way ANOVA.

Because post-transplant both self-renewal and differentiation were suppressed in the absence of CXCR4, we evaluated the early expansion of satellite cell-derived progenitors. We treated 3 month old female *Cxcr4* ^FL/FL^; *Pax7*-creERT2; *Pax7*-ZsGreen mice with five daily doses of TMX IP and on day 4 performed unilateral CTX injury of the left TA muscle. CTX injury causes hypertrophy in WT mice and accordingly, 1 month later, TA muscles of *Cxcr4* WT mice had significantly increased in mass, while *Cxcr4* KO muscles were not significantly increased and were significantly smaller (*p* < 0.001) than the injured WT (Figure 2A). We quantified the number of *Pax7*-ZsGreen cells by FACS in a subset of injured animals and found a significant failure of the satellite cells from *Cxcr4* KO to expand in response to injury, unlike the control which responded robustly to the injury by doubling the number of ZsGreen cells in the injured limb compared to the uninjured (Figures 2B,C).

### Histological abnormalities after double injury in *Cxcr4*-mutant muscle

Because previous work failed to detect histological damage after a single injury in the *Cxcr4 Pax7*-cre-driven mutant (Lahmann et al., 2021), we treated mice with TMX IP for 5 days, placed on TMX chow to minimize escapers, and subjected TA muscles to two bouts of CTX injury, 3 weeks apart (Supplementary Figure S2E). 28 days after the second injury *Cxcr4*-KO ^PAX7^ muscle showed vacuolar degeneration together with smaller myofibers (Figures 2D–F). These data suggest that *Cxcr4* is necessary in satellite cells for optimal regenerative response, particularly to severe injury.

### Endothelial cells and fibroadipogenic progenitor are the primary sources of Sdf1α in skeletal muscle.

Activation is associated with early cytological changes in the inflammatory and fibroadipogenic progenitor (FAP) compartments of skeletal muscle (Joe et al., 2010). To determine what cell types are producing SDF1α, the ligand for CXCR4, we sorted mononuclear cells from steady state and injured skeletal muscle into hematopoietic (CD45^+^), endothelial (CD31^+^), myogenic (ITGA7^+^) and FAP (PDGFRΑ^+^) components and investigated *Sdf1α* expression by RTqPCR (Supplementary Figure S3D). Almost all of the *Sdf1*α message was present in two cell types: endothelial cells and FAPs, and among these, more abundant in endothelial cells (Figure 2G). Interestingly, while in endothelial cells, *Sdf1α* expression fluctuated after injury (down at day 2, significantly elevated at day 4), it was fairly constant at these times in post-injury in FAPs.

### Satellite cell numbers decline early after injury.

We next investigated at what stage the difference in satellite cell numbers after injury could be detected. Using the same unilateral injury regimen, we analyzed muscles 1 and 2 weeks post-injury by FACS and histology (Figure 2H). Already 1 week post-injury, there was a significant reduction in the TA mass normalized to the body weight among the *Cxcr4* KO group together with a decline in the number of ZsGreen^+^ cells in the TA. At 2 weeks post injury, although muscle mass caught up somewhat, the satellite cells failed to expand and were still significantly different between WT and KO (Figures 2I–L and Supplementary Figure S2G). Histology demonstrated poor regeneration, evident by widening of the interstitial space, vacuolar degeneration, myofiber necrosis and accumulation of interstitial cells (Supplementary Figure S2F) in the KO at both 1 and 2 weeks post-injury. Furthermore, Sirius Red Fast Green staining of the TA sections revealed a widening of the endomysium, with greater numbers of smaller fibers and a trend toward greater collagen/ECM deposition in the *Cxcr4* KO group (Supplementary Figures S2H,I).

### CXCR4 regulates satellite cell maintenance

**FIGURE 3:**
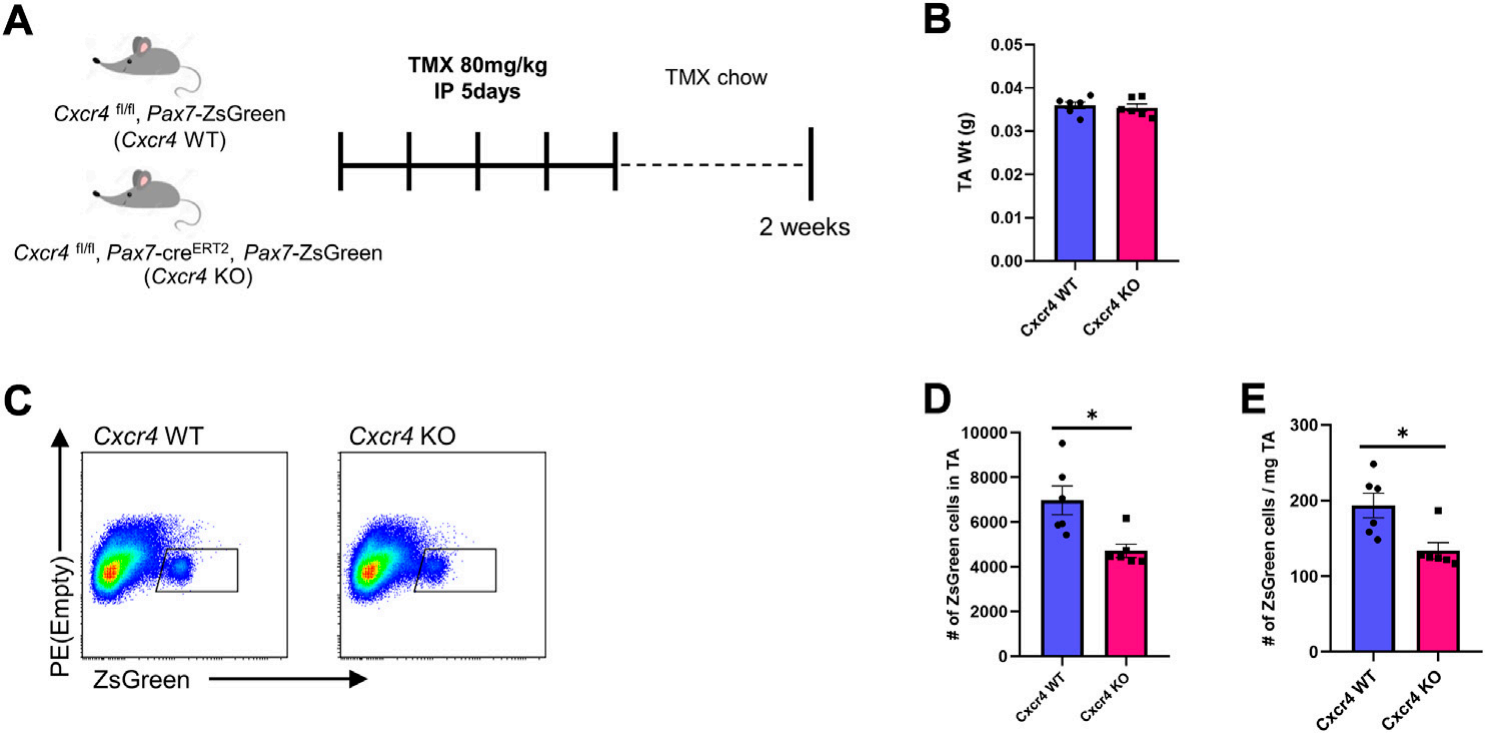
CXCR4 is necessary for satellite cell maintenance. **(A)** Schematic for Tamoxifen treatment and time of analysis. **(B)** TA mass normalized to body weight in *Cxcr4* WT and KO mice. **(C)** Representative FACS plots from *Cxcr4* WT and KO groups showing total number of ZsGreen^+^ cells in the TA muscle. **(D)** Total number of ZsGreen^+^ cells in the TA from CXCR4 WT (*n* = 3) and KO (*n* = 3) mice. **(E)** Total number of ZsGreen^+^ cells per mg TA mass from *Cxcr4* WT and KO mice. Data are presented as mean ± SEM, *p < 0.05 by *t*-test.

The defective satellite cell expansion after injury in the absence of the CXCR4 led us to investigate whether CXCR4 plays any role in steady state muscle. We treated *Cxcr4* ^FL/FL^; *Pax7*-creERT2; *Pax7*-*ZsGreen* mice with five doses of TMX IP then maintained them on TMX chow for 6 weeks (Supplementary Figure S3A) and evaluated skeletal muscles from the hindlimbs, forelimbs and diaphragm. Muscle mass and histology was not significantly altered (Supplementary Figure S3B,C), with only modest and non-significant declines in the normalized diaphragm mass, and to an even lesser extent, the TA. In contrast to whole muscle, the satellite cell population showed a degree of dependence on *Cxcr4* even in the steady state, with *Pax7*-ZsGreen^+^ cell numbers declining in the TA within 2 weeks of TMX treatment (Figure 3). As an additional control, we compared ZsGreen^+^ cells in the hind limb muscles of the *Cxcr4* ^FL/FL^; *Pax7*-ZsGreen; *Pax7*-creERT2 mice not treated with TMX to mice lacking the *Pax7*-creERT2 transgene, and found no difference in the frequency of satellite cells, demonstrating that the cre transgene has no effect in the absence of tamoxifen (Supplementary Figure S4D).

### CXCR4 regulates early satellite cell proliferation *in vitro*

**FIGURE 4:**
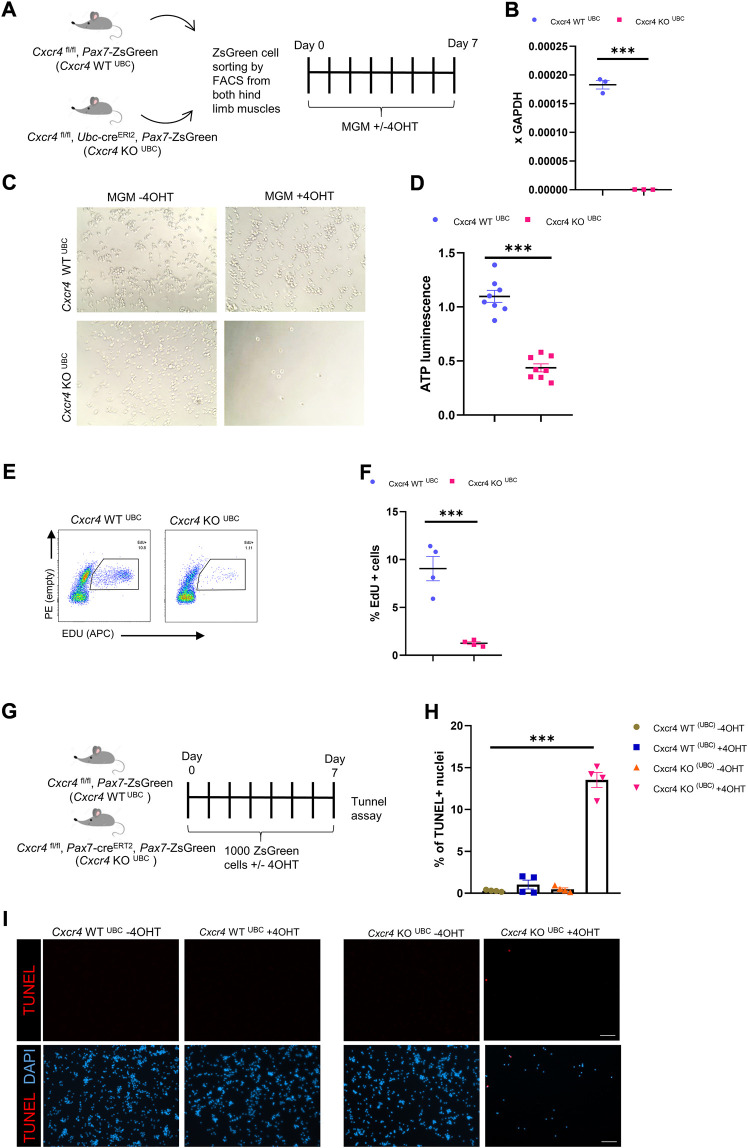
CXCR4 regulates early proliferation of satellite cells. **(A)** Schematic for Tamoxifen treatment and time of analysis. **(B)** RTqPCR for expression of Cxcr4 in myoblasts from Cxcr4^FL/FL^; Ubc-creERT2; Pax7-ZsGreen mice (KO^UBC^) (*n* = 3) or Cxcr4 ^FL/FL^; Ubc-creERT2; Pax7-ZsGreen controls lacking cre (WT^UBC^) (*n* = 3), treated with 4OHT. **(C)** Representative bright field images for myoblasts from Cxcr4-KO^UBC^ or WT^UBC^ +/- 4OHT. **(D)** Cell growth assay measuring ATP luminescence in myoblasts from Cxcr4-KO^UBC^ (*n* = 3) or WT^UBC^ (*n* = 3) +/- 4OHT. Results are normalized to untreated. **(E)** Representative FACS plots for EdU staining of myoblasts 7 days in culture from Cxcr4-WT^UBC^ +4OHT (*n* = 3) and Cxcr4 KO^UBC^ +4OHT (*n* = 3). **(F)** Frequency of EdU + cells for replicates of the cells shown in E. **(G)** Schematic for the in vitro analysis of apoptosis. **(H)** Frequency of TUNEL + nuclei in Cxcr4-WT^UBC^ and KO^UBC^ cultured satellite cells ± 4OHT. **(I)** Representative immunofluorescence staining for TUNEL in Cxcr4-WT^UBC^ and Cxcr4-KO^UBC^ +/- 4OHT scale bar = 100 µm. Note also the low cell number in KO^UBC^ + 4OHT. Data presented as mean ± SEM, ***p < 0.001 by t-test and two-way ANOVA.

**FIGURE 5:**
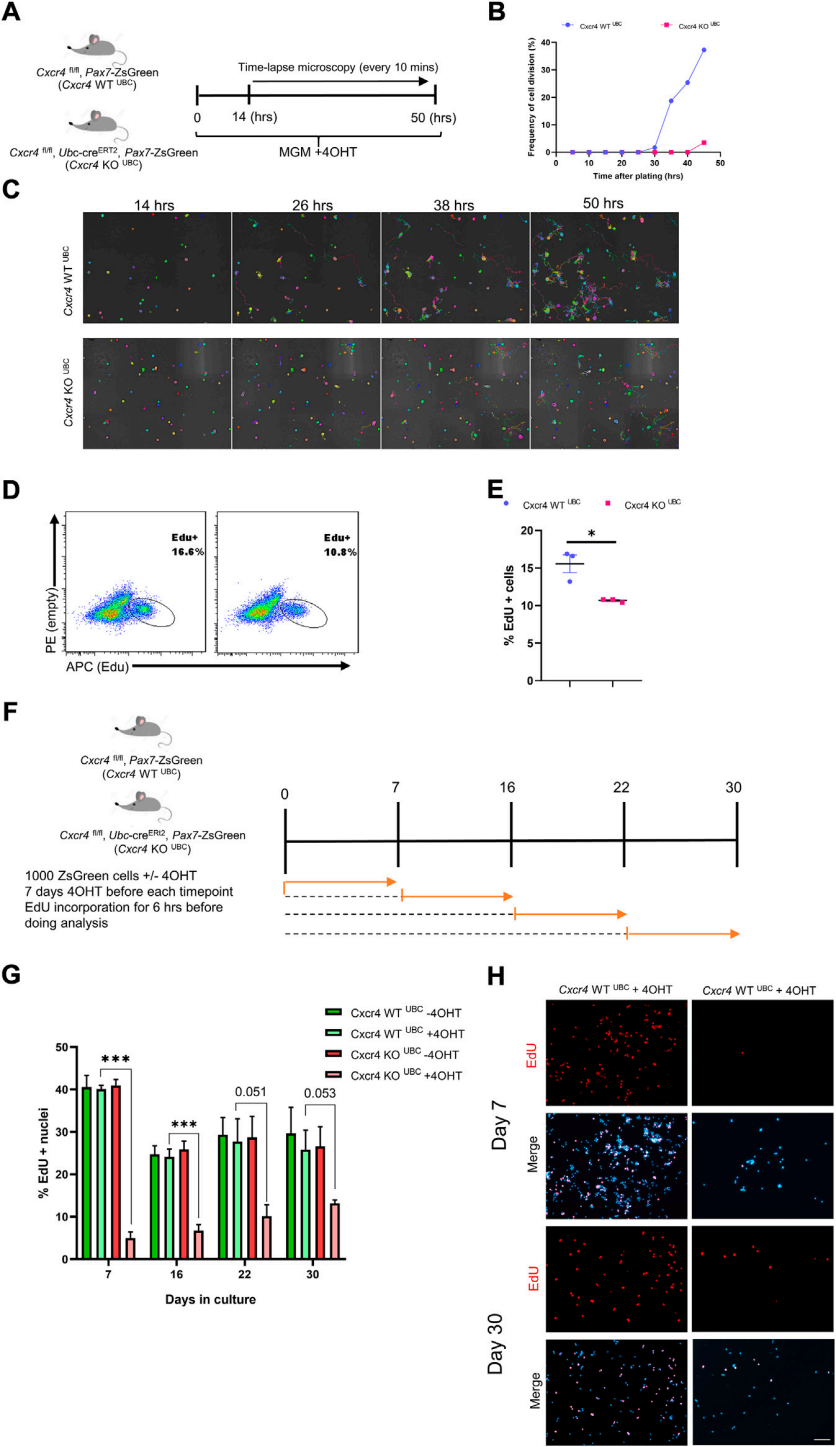
Differential dependence of satellite cells vs. established myoblasts on CXCR4. **(A)** Schematic for time lapse experiment measuring time to first division. **(B)** Frequency of satellite cells having undertaken first division over time. **(C)** Representative images tracing satellite cell mobility during time lapse imaging. Colors were randomly assigned by the Nikon Element software for every Binary Particle in the Time lapse movie. **(D)** Representative FACS plots for EdU staining of established myoblasts (6 weeks in culture) from Cxcr4-KO^UBC^ (*n* = 3) and Cxcr4-KO^UBC^ (*n* = 3) mice. Established myoblasts were treated with 4OHT for 1 week, and treated with EdU for the last 6 h of the final day. **(E)** Quantification of EdU+ cells for the experiment shown in D. **(F)** Schematic for testing proliferation dependence on CXCR4 in cultured satellite cells over time. **(G)** Frequency of EdU+ nuclei in myoblasts tested after 4OTH addition at various times over 30 days in culture. Data are presented as mean ± SEM, ***p < 0.001 by *t*-test. **(H)** Representative EdU incorporation at day 7 and day 30 in the experiment outlined in F. Scale bar = 100 µm.

To further characterize the satellite cell impairment, we studied the behavior of freshly isolated satellite cells in culture. *Cxcr4* ^FL/FL^, *Pax7*-cre^ERT2^+/-, *Pax7*-ZsGreen mice and cre-neg controls were treated with tamoxifen (5 IP injections), and hind limb satellite cells plated into growth medium (Supplementary Figure S4A). Cells from mice lacking cre grew out much more efficiently (Supplementary Figure S4B); furthermore, adding 4OHT to the medium selectively suppressed outgrowth of cells from cre+ mice, suggesting that escapers contributed to the residual growth of the FL/FL cells. Because *Pax7* is downregulated upon activation, the *Pax7-creERT2* allele is not expressed efficiently *in vitro*. To study deletion at later time points *in vitro*, we made use of the *Ubc-cre* allele. Short term cultures of satellite cells from *Cxcr4* ^FL/FL^, *Ubc*-cre^ERT2^+/-, *Pax7*-ZsGreen mice exposed to 4OHT (4-hydroxy tamoxifen) *in vitro* efficiently lost *Cxcr4* expression (Figures 4A,B, Supplementary Figure S2B), and failed to proliferate, morphologically (Figure 4C), quantitatively by ATP assay (Figure 4D), and by EdU incorporation (Figures 4E,F)*.* A large fraction of cells in the KO arm + 4OHT were undergoing apoptosis by the 7 day time point, as shown by TUNEL (Figures 4G–I). Time lapse imaging showed that the great majority of cells had not undertaken their first division by 50 h post-plating (Figures 5A,B), and furthermore showed greatly reduced cytomobility (Figure 5C, Supplementary Figure S4C, Supplementary Video S1).

We then tested whether CXCR4 was necessary for the proliferation of established myoblasts. When cultures obtained from *Cxcr4* ^FL/FL^; *Ubc*-creERT2 satellite cells were passaged for 6 weeks as myoblasts and then exposed to 4OHT, cells continued to grow robustly, although they showed a modest but statistically significant decline in EdU incorporation suggesting a slightly longer cell cycle (Figures 5D,E). To dissect the temporal necessity of CXCR4, we set up a new experiment in which *Cxcr4* WT ^UBC^ and *Cxcr4* KO ^UBC^ cells were grown in myogenic growth medium and treated with 4OHT at various intervals (day 0, 7, 16 and 22), given 7 days of further growth to allow complete deletion of *Cxcr4*, treated with EdU for 6 h (Figure 5F). This revealed a much greater *Cxcr4*-dependence in the earliest cell divisions than in later divisions, with EdU incorporation in the absence of *Cxcr4* gradually increasing with age of culture (Figures 5G,H and Supplementary Figure S5A). Moreover, we assessed the colony-forming activity of satellite cells in the absence of CXCR4. Single satellite cells from *Cxcr4* -WT ^UBC^ and *Cxcr4*-KO ^UBC^ mice were plated in 96-well dishes + 4OHT and colonies were assessed 8 days later (Ippolito et al., 2012) (Supplementary Figure S5B). Both the ability of the cells to form colonies *in vitro* and the mean colony sizes were reduced in the *Ubc*-cre group compared to cre-negative controls (Supplementary Figures S5C,D).

### Transcriptional profile suggests defective activation in the absence of CXCR4.

**FIGURE 6:**
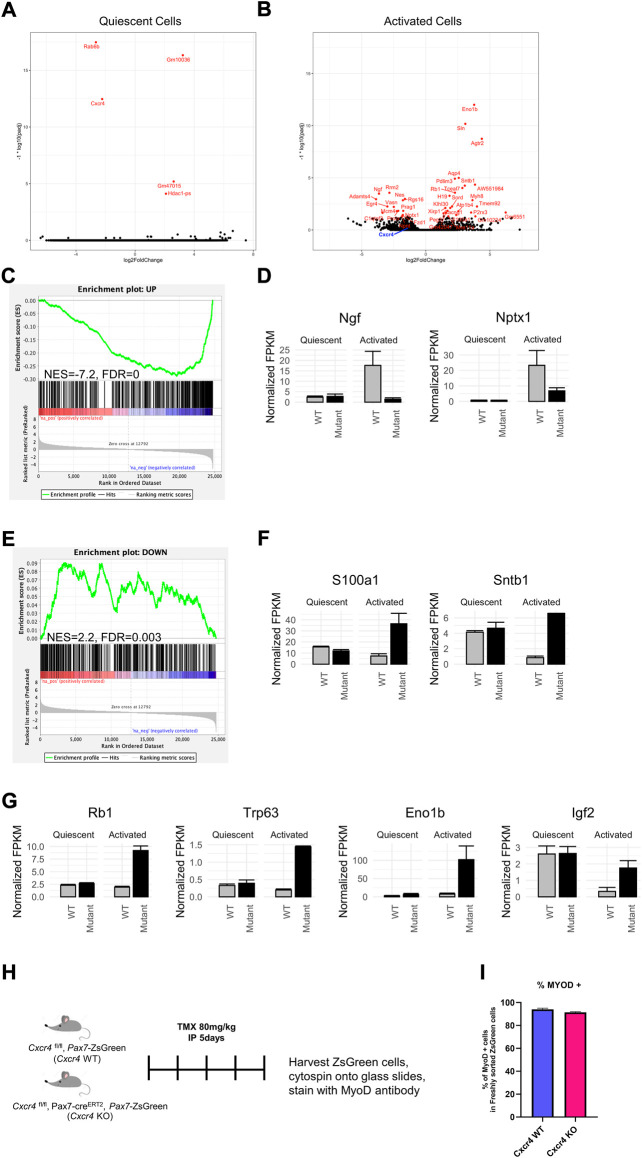
CXCR4 mediates the satellite cell early activation but not quiescence transcriptional profile. **(A)** Volcano plot showing differentially expressed genes in freshly isolated Cxcr4-KO ^PAX7^ or WT satellite cells from steady state muscle. The -log10 p-value and log2 fold changes of genes in Cxcr4 knockout cells compared to controls are plotted such that genes that are more highly significant are higher on the y-axis. **(B)** Volcano plot showing differentially expressed genes in Cxcr4-KO ^PAX7^ or WT satellite cells undergoing activation (overnight culture in MGM). **(C)** Gene set enrichment analysis (GSEA) for changes between Cxcr4-KO ^PAX7^ and WT compared to wild type in the activated state, using a set of genes previously determined to be upregulated with activation (Machado et al., 2017). **(D)** Barplots of example genes that were within the activation-UP gene set with significant p-values (Benjamini–Hochberg adjusted p-value < 0.05). Note the lack of up-regulation in the mutant. **(E)** GSEA for Cxcr4-dependent transcriptional changes using the set of genes determined to be downregulated with activation by (Machado et al., 2017). Note the failure of down-regulation in the mutant. **(F)** Barplots of example genes that were within the activation-DOWN gene set with significant p-values (Benjamini–Hochberg adjusted p-value < 0.05). **(G)** Examples of genes dysregulated in the Cxcr4 mutant that may be associated with cell cycle arrest. **(H)** Schematic of analysis of translation of MYOD1 transcript in freshly isolated cells of Cxcr4 KO ^PAX7^ or WT ^PAX7^. **(I)** Quantification of MYOD1 positive satellite cells immediately after muscle digestion and FACS isolation.

To better understand the mechanism of *Cxcr4* activity in satellite cells, we performed transcriptional profiling. In the steady state, satellite cells lacking *Cxcr4* showed virtually no statistically significant differences in gene expression (Figure 6A). We next profiled activated satellite cells, by studying gene expression after isolation and overnight culture in growth medium. This revealed a large set of differentially expressed genes (Figure 6B), demonstrating that the primary activity of *Cxcr4* signaling as it relates to transcription is in activated as opposed to quiescent satellite cells (Supplementary Tables S2A,B). By inspection, we noted that many genes associated with early activation were diminished in expression levels in *Cxcr4* KO activated satellite cells, therefore we evaluated a set of previously-defined genes whose expression changes upon satellite cell activation (Machado et al., 2017). GSEA revealed a large subset of genes normally upregulated with activation to be showing defective upregulation in the *Cxcr4* KO (Figures 6C,D). Genes normally downregulated with activation also showed a statistically significant negative correlation, however the effect on these genes was much less pronounced than the effect on the upregulated set (Figures 6E,F). In addition to the misregulation of genes normally observed in activation, we also observed upregulation of the genes such as *Rb1* and *Trp63* that may block cell cycle progression and compromise satellite cell activation (Figure 6G). Paradoxically, we also noted upregulation of genes that can have growth-promoting effects, including the enolase paralog *Eno1b* and insulin growth factor 2 (*Igf2*). Together these data demonstrate clearly that activation is impaired in the *Cxcr4* KO and suggest possible mechanisms that could be tested in future studies.

Activation doubtlessly involves multiple pathways, and is a multi-step process. One of the earliest events is the expression of certain proteins whose RNA is already present but not translated (Crist et al., 2012). One very clear example of this can be seen by immunostaining for MYOD1. In histological sections, satellite cells are negative for MYOD1 protein, however as soon as they are isolated, a process that involves enzyme incubation for at least an hour at 37°C, they become MYOD1 protein+. To evaluate this very early stage in activation, we sorted satellite cells from WT and Pax7-driven *Cxcr4* KO and spun them onto glass slides for immunostaining (Figure 6H). MYOD1 was present in almost all cells, in both the WT and KO arms (Figure 6I). This demonstrates that CXCR4 activity has no bearing on this earliest step in activation, and that its major activity is limited to a window of time between when cells become MYOD1+ and the first cell division.

## Discussion

CXCR4 is best known for its role in chemotaxis, and indeed its major role in embryonic myogenesis relates to migration of myogenic progenitors (Vasyutina et al., 2005; Yusuf et al., 2005; Yusuf et al., 2006; Rehimi et al., 2010). While satellite cells are not migratory between muscles (Schultz et al., 1986), they are motile along the fiber in response to activation (Gu et al., 2016), and they do occupy a stereotypical position in relation to the endothelial network (Verma et al., 2018), suggesting a requirement for chemotactic/sensing systems, and indeed our time lapse study demonstrated much reduced motility of *Cxcr4*-null satellite cells, suggesting a potential role for CXCR4 in satellite cell movement and orientation. Regarding roles in more regeneration-centric functions, such as activation, proliferation and differentiation, a recent study had shown that while *Cxcr4* mutation enhanced the regeneration phenotype of c-met mutation in satellite cells, deletion of *Cxcr4* alone had no discernable effect on the extent of regeneration after injury (Lahmann et al., 2021). We were therefore surprised at the extent of difference between *Cxcr4*-KO and -WT cells in our initial transplantation assay, an assay that is much more dependent on survival, proliferation, and differentiation than on chemotaxis. CXCR4 was necessary both for the ability of satellite cells to repopulate the satellite cell pool, as well as for cells to be able to contribute efficiently to the population of new fibers. The likely reason this had not been appreciated previously is that the degree of regenerative demand on a per cell basis is much higher in a 300 cell transplant into an injured irradiated TA muscle than in a singly CTX-injured muscle. In addition, the tremendous potential of “escapers”, satellite cells that escape recombination, to suppress satellite cell specific deletion phenotypes has been recognized (Gunther et al., 2013; von Maltzahn et al., 2013), which motivated the addition of tamoxifen chow following the tamoxifen injections, and represents a difference in methodology compared to the Lahmann study. In the current study, we found that after a single CTX injury, which normally leads to substantial hypertrophy accompanied by a doubling of the satellite cell population, there was virtually no hypertrophy or expansion in the satellite cell pool if *Cxcr4* had been deleted in PAX7+ cells. Furthermore, in the more stringent double-CTX injury model, fiber size was significantly reduced in the regenerated muscles in which *Cxcr4* was deleted in PAX7+ cells.

Evaluating regenerating muscle at earlier time points revealed that the defect in regeneration was already manifest in *Cxcr4* mutants at the first week post-injury, both in terms of muscle mass and in the significant drop in numbers of the satellite cell pool. This suggested that CXCR4 signaling was most critical in the early stages of muscle regeneration, possibly for the cell divisions necessary to produce the large number of myoblasts that will differentiate into the mass of newly-generated muscle. To test this idea, we investigated the *Cxcr4*-dependence of proliferation by deleting *Cxcr4* in cells proliferating *in vitro*, comparing freshly isolated satellite cells, passaged satellite cells and established myoblasts. While cell division was impaired to some degree at all stages, demonstrating that CXCR4 signaling promotes cell cycle transit generally in myogenic progenitors, the impairment was more severe the younger the culture was. When *Cxcr4* was deleted prior to plating, most *Cxcr4*-deleted satellite cells failed to divide at all within the time frame of the study, in which almost all WT cells had undertaken their first division. Cell division represents the culmination of the activation process. To assess the earliest known event, translation of mRNAs sequestered in RNP granules, we assessed MYOD protein in freshly isolated *Cxcr4*-deleted satellite cells. This earliest step occurs during the enzyme digestion stage, and we found no difference in MYOD positivity between CXCR4 WT and KO satellite cells, with uniform staining in both populations.

To understand what specifically might be going awry slightly later in the activation process, we performed RNA-seq on freshly isolated satellite cells after a short period of culture, well prior to first division. In contrast to RNA-seq on *Pax7*-ZsGreen cells immediately after FACS isolation, in which very few gene expression differences could be identified, RNA-seq on activated cells showed many differences, most particularly a highly significant enrichment for changes in genes normally upregulated by activation (Machado et al., 2017). While not all genes normally upregulated by activation were lower in the *Cxcr4* mutant, a clear subset of these genes was dependent on *Cxcr4* for their normal upregulation with activation. These data, together with the fact that most *Cxcr4* mutant satellite cells fail to divide, indicate that *Cxcr4* plays a key role in satellite cell activation. It is interesting to note that the alarmin HMGB1, which is known to promote signaling through CXCR4 (Schiraldi et al., 2012), was recently implicated as necessary for the G-alert state of several cell types, including satellite cells, in response to fracture injury (Lee et al., 2018). It would therefore be interesting to evaluate the degree to which entry into G-alert is affected in the *Cxcr4* KO.

The SDF1α/CXCR4 axis has also been implicated in cell survival in a number of studies (Jiang et al., 2018; Dalal et al., 2020; Aronovich et al., 2021; Lahmann et al., 2021). We observed an increase in TUNEL+ cells when *Cxcr4* was deleted in satellite cells cultured *in vitro*, suggesting a role for survival in addition to proliferation. However, given that the TUNEL staining was done 7 days after deletion, and in cells that were basically not dividing, the elevated apoptosis may represent a secondary effect.

To determine the source of the signal to which CXCR4 is responding, we sorted various subcomponents of the muscle mononuclear fraction and evaluated their expression of *Sdf1α*. While *Sdf1α* could be detected in all fractions, it was robustly expressed in two cell types: endothelial cells and FAPs. Although well expressed at all time points by endothelial cells, we found that the *Sdf1α* expression was reduced at 2 days post-injury, but significantly upregulated at 4 days post-injury. In FAPs, its expression did not change over these time points. Given the critical early necessity of *Cxcr4*, it was somewhat surprising to see *Sdf1α* expression trend downwards 2 days after injury in endothelial cells, thus since FAPs are known to promote proliferation of satellite cells (Joe et al., 2010), it may be through interaction with FAPs that this signal is primarily mediated in the earliest time points after injury. Notwithstanding, it would be interesting to determine the extent to which the SDF1α/CXCR4 axis affects the positioning of quiescent satellite cells in the vicinity of endothelial cells. The role of Sdf1α in endothelial cells and FAPs, both after injury or during steady state, will await cell type-specific deletion studies.

In summary, these studies demonstrate that maintained expression of *Cxcr4* from the fetal stage into the adult phase has functional consequences beyond chemotaxis. Using a protocol that involves following tamoxifen-mediated satellite cell-specific deletion with continued tamoxifen provision *via* food in order to suppress potential escapers, we find that *Cxcr4* is critically necessary for appropriate activation and for early proliferation of satellite cells and their immediate progeny, and thereby necessary for normal skeletal muscle regeneration.

## Data Availability

The sequence data presented in the study are deposited in the GEO repository, accession number GSE205015.
