# Supplementary material for: The chemokine receptor CXCR4 regulates satellite cell activation, early expansion, and self-renewal, in response to skeletal muscle injury
Source: Front Cell Dev Biol. 2022 Sep 22;10:949532. doi: 10.3389/fcell.2022.949532 (PMC9536311; doi:10.3389/fcell.2022.949532)
Supplement: Supplementary file 1 [file DataSheet2.PDF]

Supplementary Table 1

Antibody name	Identifiers	Dilution	Source
PE-Cy7 rat ant-mouse CD31	Clone 390	1:100	BD Bioscience San Diego, CA, USA
PE-Cy7 rat anti-mouse CD45	Clone 30-F11	1:100	BD Bioscience San Diego, CA, USA
Biotin rat anti-mouse CD106	Clone 429 (MVCAM.A)	1:100	BD Bioscience San Diego, CA, USA
PE Streptavidin	AB_11154598	1:100	BD Bioscience San Diego, CA, USA
ITGA7 647	Clone R2F2	1:100	AbLab Vancouver, B.C., Canada
FITC rat anti-mouse CD31	Clone MEC13.3	1:100	BioLegend San Diego, CA, USA
Biotin Rat anti-Mouse CD184	Clone 2B11 /CXCR4	1:100	BD Bioscience San Diego, CA, USA
PE rat anti-mouse CD140A	Clone APA5	1:100	BD Bioscience San Diego, CA, USA
Rabbit polyclonal antibody to dystrophin	Ab15277	1:250	Abcam, Cambridge, MA, USA
mouse monoclonal antibody to laminin	clone LAM-89	1:500	Sigma Saint Louis, MO, USA
Alexa Fluor 555 goat anti-rabbit IgG	A32727	1:1000	Life Technologies, Grand Island, NY, USA
Alexa Fluor 488 goat anti-mouse IgG	A-11008	1:1000	Life Technologies, Grand Island, NY, USA

Supplementary Table 2

A) Quiescent Satellite Cells

Gene ID	Gene Symbol	Mean Counts	Log2 Fold Change	BH Adjusted P-value	Gene Type	Chromosome
ENSMUSG00000032549	Rab6b	242.0541151	-2.652276895	3.39E-18	protein_coding	chr9
ENSMUSG00000058064	Gm10036	173.619661	3.229804422	4.72E-17	processed_pseudogene	chr18
ENSMUSG00000045382	Cxcr4	536.9952471	-2.226048592	3.45E-13	protein_coding	chr1
ENSMUSG00000112907	Gm47015	98.97380401	2.609127939	6.44E-06	lincRNA	chr10
ENSMUSG00000061062	Hdac1-ps	81.90591805	2.087531963	7.67E-05	processed_pseudogene	chr17

B) Activated Satellite Cells

Gene ID	Gene Symbol	Mean Counts	Log2 Fold Change	BH Adjusted P-value	Gene Type	Chromosome
ENSMUSG00000059040	Eno1b	1163.37753	3.772562354	9.89E-13	protein_coding	chr18
ENSMUSG00000042045	Sln	1029.512476	3.082838244	6.66E-11	protein_coding	chr9
ENSMUSG00000068122	Agtr2	257.6269175	4.369312317	1.80E-09	protein_coding	chrX
ENSMUSG00000024411	Aqp4	1065.038606	2.570027569	1.02E-05	protein_coding	chr18
ENSMUSG00000031636	Pdlim3	2055.958201	2.267391522	1.21E-05	protein_coding	chr8
ENSMUSG00000038112	AW551984	238.6500717	3.836723371	4.53E-05	protein_coding	chr9
ENSMUSG00000060429	Sntb1	223.6204837	3.040607356	5.65E-05	protein_coding	chr15
ENSMUSG00000079428	Tceal7	415.0139617	2.852941344	9.53E-05	protein_coding	chrX
ENSMUSG00000022105	Rb1	378.6591864	2.273772861	0.00026677	protein_coding	chr14
ENSMUSG00000020649	Rrm2	252.5553245	-2.838184262	0.000272864	protein_coding	chr12
ENSMUSG00000027859	Ngf	157.8282959	-3.65020954	0.00033126	protein_coding	chr3
ENSMUSG00000000031	H19	18476.17635	1.851099271	0.000531096	lincRNA	chr7
ENSMUSG00000004891	Nes	2834.00909	-1.628156087	0.001050803	protein_coding	chr3
ENSMUSG00000006403	Adamts4	193.0446583	-3.878716779	0.001133616	protein_coding	chr1
ENSMUSG00000055775	Myh8	114.3399377	3.640490308	0.001398961	protein_coding	chr11
ENSMUSG00000026475	Rgs16	1032.347117	-1.787261663	0.001427632	protein_coding	chr1
ENSMUSG00000027227	Sord	612.1957795	2.04555526	0.005616703	protein_coding	chr2
ENSMUSG00000071341	Egr4	124.9540798	-2.995909583	0.005616703	protein_coding	chr6
ENSMUSG00000075610	Tmem92	87.60648942	3.991674583	0.005616703	protein_coding	chr11
ENSMUSG00000039646	Vasn	517.8420636	-2.493970926	0.006181452	protein_coding	chr16
ENSMUSG00000026308	Klhl30	405.4432618	1.909426366	0.007777711	protein_coding	chr1
ENSMUSG00000079243	Xirp1	772.3863622	1.524965477	0.007790664	protein_coding	chr9
ENSMUSG00000027611	Procr	993.6171727	-2.146518072	0.014776426	protein_coding	chr2
ENSMUSG00000050271	Prag1	527.8599496	-1.75969472	0.014776426	protein_coding	chr8
ENSMUSG00000020722	Cacng1	678.3387171	1.696438029	0.014776426	protein_coding	chr11
ENSMUSG00000022673	Mcm4	211.1670569	-2.268867356	0.015896043	protein_coding	chr16
ENSMUSG00000016327	Atp1b4	203.7028832	2.540651466	0.016010158	protein_coding	chrX
ENSMUSG00000027071	P2rx3	80.08209613	3.53407032	0.021288571	protein_coding	chr2
ENSMUSG00000110631	Gm42047	1068.305372	1.581872001	0.021288571	lincRNA	chr8
ENSMUSG00000116659	Gm6551	54.05359062	6.229026475	0.021288571	unprocessed_pseudogen	chr16
ENSMUSG00000058914	C1qtnf3	82.46108478	-3.368797657	0.022690323	protein_coding	chr15
ENSMUSG00000002265	Peg3	854.3981095	1.380794811	0.025363425	protein_coding	chr7
ENSMUSG00000044080	S100a1	172.0726244	2.372469681	0.036537329	protein_coding	chr3
ENSMUSG00000025582	Nptx1	888.1188837	-1.789683877	0.036537329	protein_coding	chr11
ENSMUSG00000044674	Fzd1	314.3563142	-1.809495116	0.041973752	protein_coding	chr5
ENSMUSG00000040998	Npnt	310.2917794	-1.856277737	0.049678715	protein_coding	chr3
ENSMUSG00000080932	Gm10224	134.575248	3.867487951	0.049678715	processed_pseudogene	chr6
ENSMUSG00000031928	Mre11a	283.1651856	1.759852704	0.049678715	protein_coding	chr9

Supplemental Table 2 Lists of genes differentially expressed between wild type and *Cxcr4* knock-out satellite cells in quiescent (A) and activated (B) states. Differentially expressed genes were defined as having a fold change greater than 2 (log2 fold change > 1 or < -1), a mean number of counts across samples greater than 10 and an adjusted p-value less than 0.05. The Benjamini-Hochberg (BH) correction was applied to the p-values to adjust for multiple tests.
