# Supplementary material for: The chemokine receptor CXCR4 regulates satellite cell activation, early expansion, and self-renewal, in response to skeletal muscle injury
Source: Front Cell Dev Biol. 2022 Sep 22;10:949532. doi: 10.3389/fcell.2022.949532 (PMC9536311; doi:10.3389/fcell.2022.949532)
Supplement: Supplementary file 3 [file DataSheet1.PDF]

Supplementary Figure 1

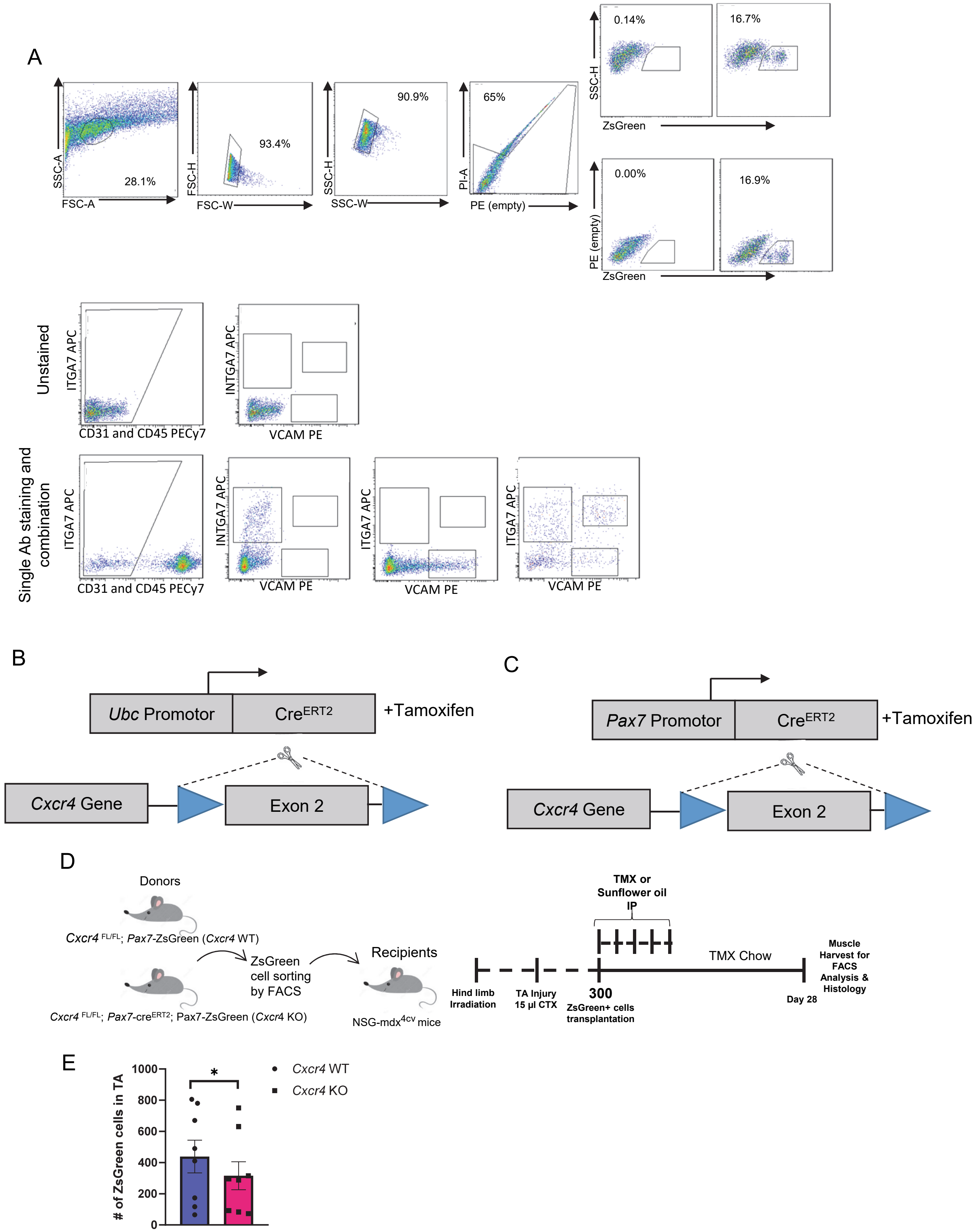

(A) Schematic of FACS gating strategies. Above, strategy for identifying ZsGreen+ cells: PI-negative cells were evaluated on SSC vs. green channels or the PE vs the green channels. Below, strategy for identifying satellite cells based on surface markers: PI-negative cells were evaluated on the APC (ITGA7) vs. PE-Cy7 (CD31 and CD45 Lineage cocktail) and the APC (ITGA7) vs. PE (VCAM). Satellite cells are Lin-neg, ITGA7+, VCAM+.

(B) Schematic for the ubiquitous cre^{ERT2} deleting exon 2 of the *Cxcr4* gene. With the administration of Tamoxifen, cre is expressed ubiquitously and exon 2, which is flanked by loxP sites, is deleted. *Pax7*-cre^{ERT2}.

(C) Schematic for the satellite cell-specific *Pax7*-cre^{ERT2} deleting exon 2 of the *Cxcr4* gene.

(D) Schematic for the transplantation assay using donor mice bearing Pax-7cre^{ERT2} . All recipients were treated with Tamoxifen IP then kept on Tamoxifen chow.

(E) Total number of ZsGreen^{+} (donor, satellite) cells in the TA muscle of the recipient NSG-mdx^{4cv} mice (n=8). Data are presented as mean±SEM, * P<0.05 by t test.

Supplementary Figure 2

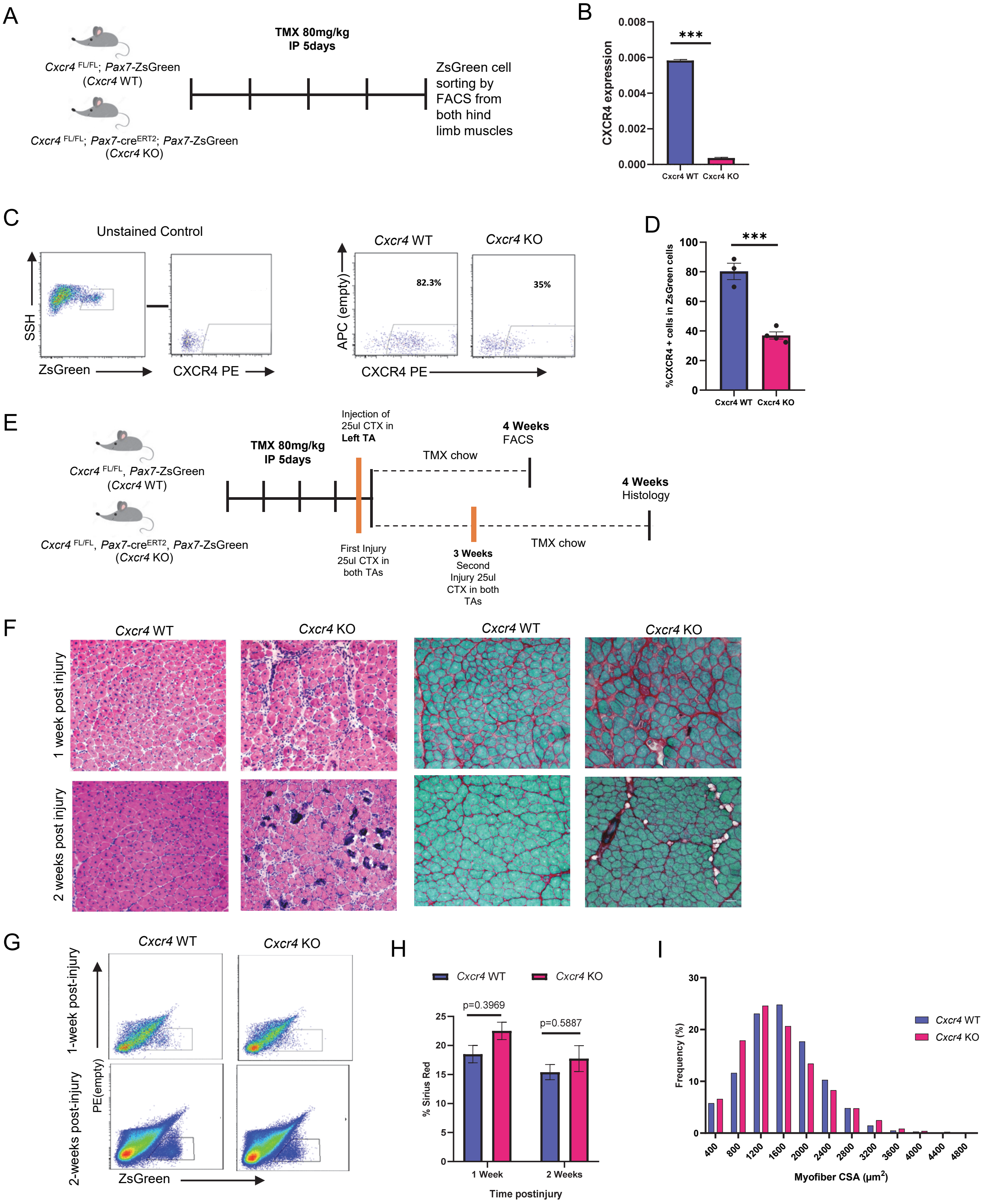

(A) Schematic for mice treatment and time of analysis.
(B) RTqPCR for *Cxcr4* expression in freshly sorted ZsGreen cells from *Cxcr4* WT (n=3) and *Cxcr4* KO (n=3) mice, 5 days after Tamoxifen injection.
(C) Representative FACS plots showing percentage of CXCR4+ cells stained with CXCR4-biotin and streptavidin-PE" in the ZsGreen+ population from *Cxcr4* WT and *Cxcr4* KO mice.
(D) Percentage of CXCR4+ cells in the ZsGreen^{+} population from *Cxcr4* WT (n=3) and *Cxcr4* KO (n=4) mice.
(E) Schematic of the injury experiments.
(F) Representative H&E (left) and Sirius Red / Fast Green (SRFG, right) staining of TA sections from *Cxcr4*-WT and *Cxcr4*-KO mice. Scale bar= 50μm.
(G) Representative FACS plots showing the number of ZsGreen+ cells in the TA of *Cxcr4* WT and *Cxcr4* KO mice.
(H) Percentage of Sirus Red-stained areas in the TA section stained with SRFG from *Cxcr4* WT (n=2) and *Cxcr4* KO (n=2) mice.
(I) Relative frequency of myofibers CSA in TA sections from *Cxcr4* WT and *Cxcr4* KO mice 2 weeks post-injury. Data are presented as mean±SEM, *** P<0.001 by t test. Data are presented as mean±SEM, *** P<0.001 by t test.

Supplementary Figure 3

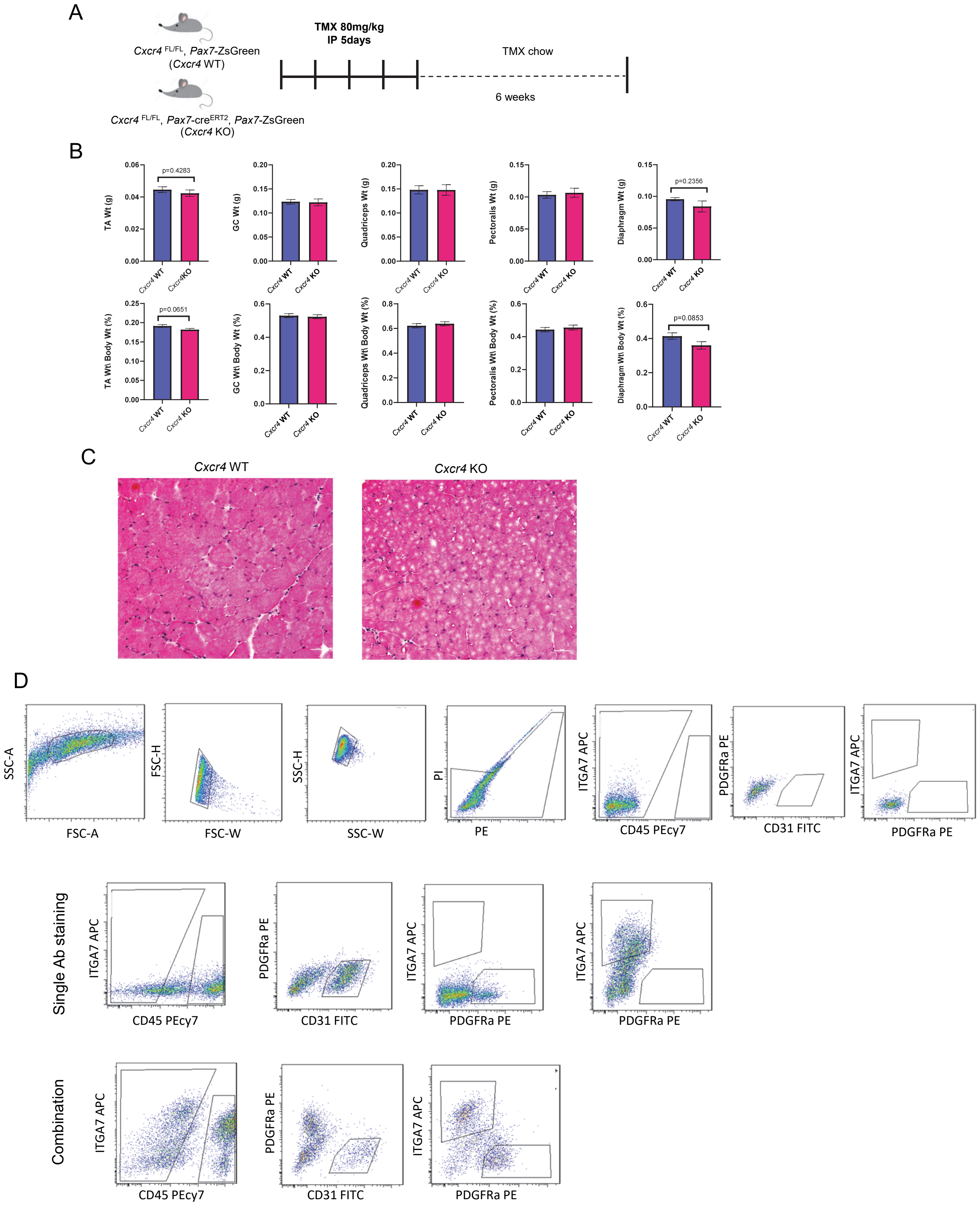

(A) Schematic for mice treatment and time of analysis.

(B) Average total and body weight-normalized mass for 5 muscle groups from *Cxcr4* WT (n=4) and *Cxcr4* KO (n=4) mice. GC – gastrocnemius.

(C) Representative images for H&E staining of quadriceps sections from *Cxcr4* WT and *Cxcr4* KO mice. Scale bar = 50μm. Data presented as mean±SEM.

(D) Schematic of FACS gating strategy for sorting the skeletal muscle niche cells. The upper panel shows the unstained sample. The PI- population was evaluated on the APC (ITGA7) and PECy7 (CD45), then the CD45- population was evaluated on the PE channel (PDGFRa) and APC (ITGA7) and FITC (CD31).

Supplementary Figure 4

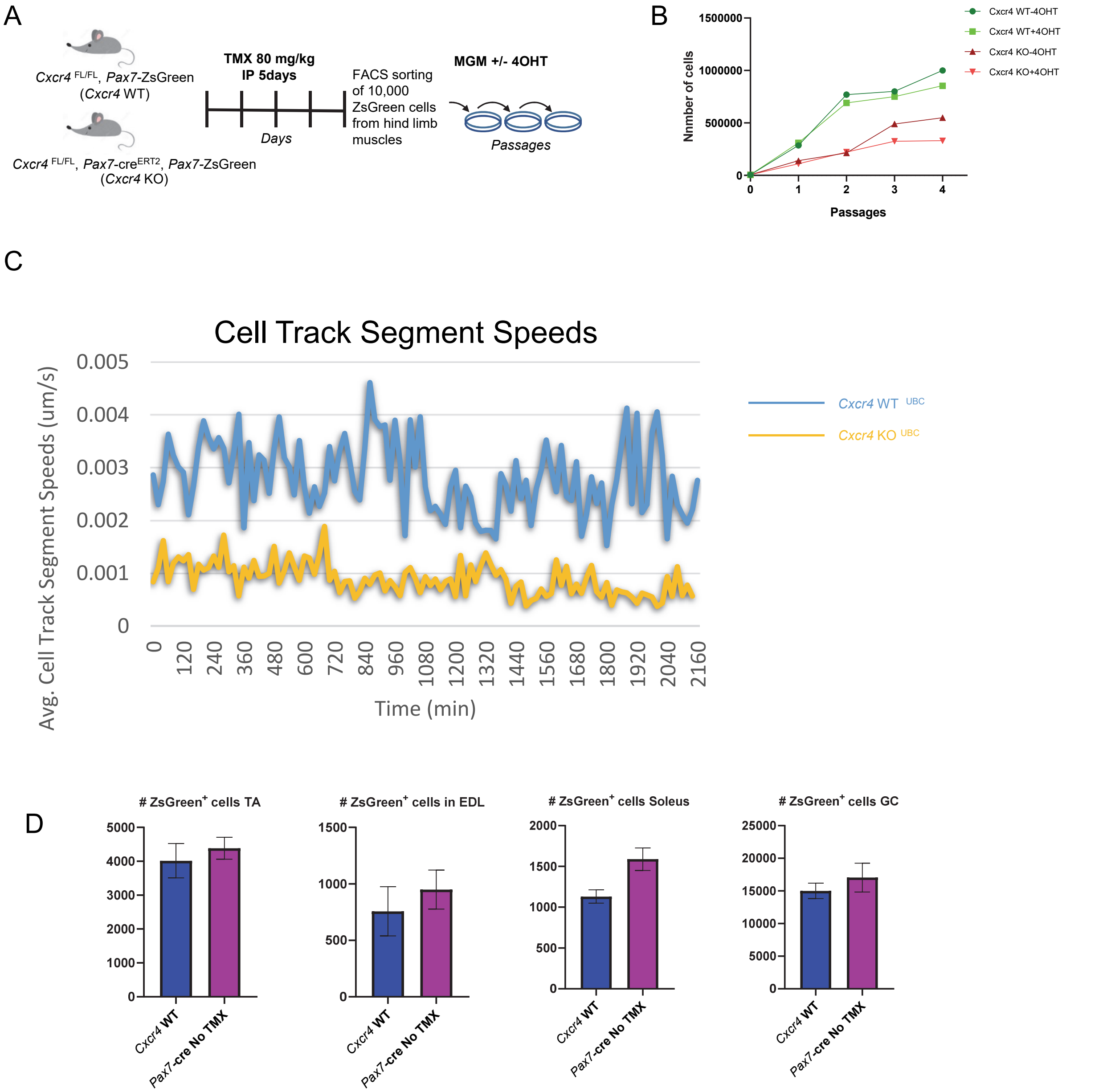

(A) Schematic for mice treatments followed by ZsGreen+ cell culture under different conditions.

(B) Growth curve showing the proliferation of the ZsGreen cells from both the *Cxcr4* WT and *Cxcr4* KO mice culture in the presence/absence of 4OHT.

(C) Chart tracing the average speed of ZsGreen+ cells from *Cxcr4* WT^{UBC} and the *Cxcr4* KO^{UBC} during time lapse live cell imaging.

(D) Analysis of the number of Pax7-ZsGreen+ cells in different hind limb muscles from Cxcr4$^{FL/FL}$; Pax7-ZsGreen either carrying or not carrying the Pax7-creERT2 transgene. Mice were not treated with tamoxifen (n=4). The lack of difference indicates that the cre insertion and any linked alleles on the insertion chromosome have no influence on satellite cell number.

Supplementary Figure 5

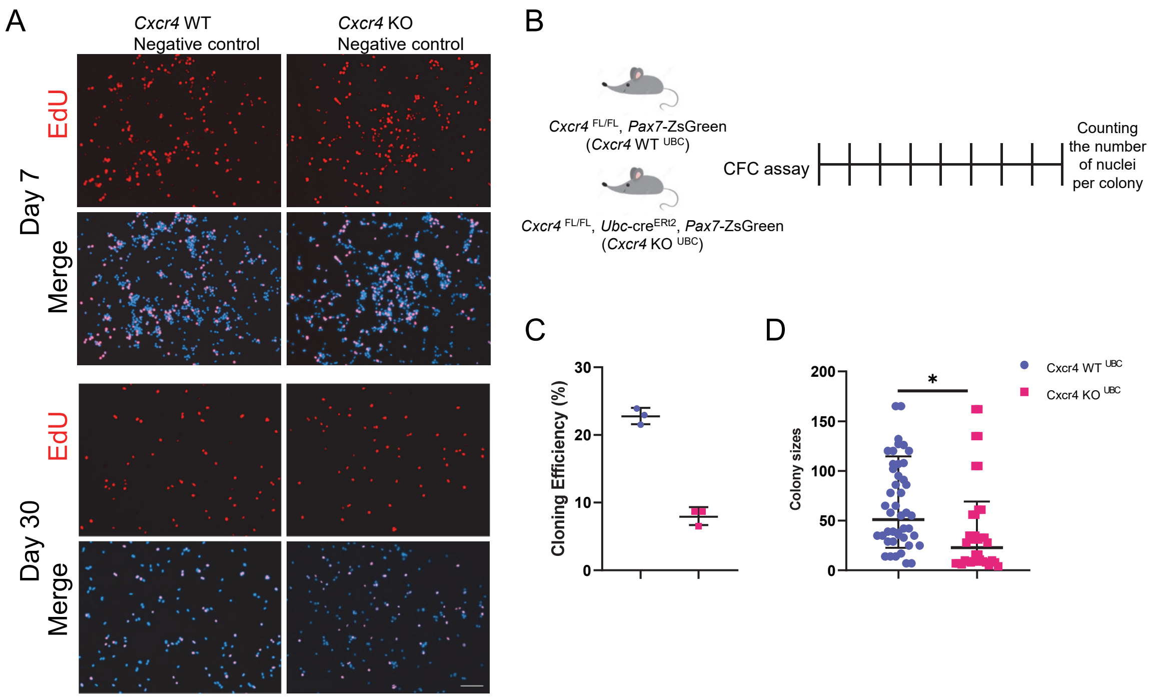

(A) Representative IF images for negative controls (absence of 4OHT) for cultured myoblasts from *Cxcr4* WT^{UBC} and the *Cxcr4* KO^{UBC} mice.

(B) Schematic for the Colony Forming Cell assay. Scale bar=100 μm.

(C) Cloning efficiency of single sorted cells from *Cxcr4* WT^{UBC} (n=3) and the *Cxcr4* KO^{UBC} (n=3) mice.

(D) Colony Sizes of day 8-cultured single cells from *Cxcr4* WT^{UBC} and the *Cxcr4* KO^{UBC} mice. Data are presented as Geometric mean±SD, * P<0.05 by t test.
